# Microglial Activation Promotes Cell Survival in Organotypic Cultures of Postnatal Mouse Retinal Explants

**DOI:** 10.1371/journal.pone.0135238

**Published:** 2015-08-07

**Authors:** Rosa M. Ferrer-Martín, David Martín-Oliva, Ana Sierra-Martín, Maria-Carmen Carrasco, María Martín-Estebané, Ruth Calvente, Sandra M. Martín-Guerrero, José L. Marín-Teva, Julio Navascués, Miguel A. Cuadros

**Affiliations:** Departamento de Biología Celular, Facultad de Ciencias, Universidad de Granada, Granada, Spain; Indiana School of Medicine, UNITED STATES

## Abstract

The role of microglia during neurodegeneration remains controversial. We investigated whether microglial cells have a neurotoxic or neuroprotective function in the retina. Retinal explants from 10-day-old mice were treated *in vitro* with minocycline to inhibit microglial activation, with LPS to increase microglial activation, or with liposomes loaded with clodronate (Lip-Clo) to deplete microglial cells. Flow cytometry was used to assess the viability of retinal cells in the explants and the TUNEL method to show the distribution of dead cells. The immunophenotypic and morphological features of microglia and their distribution were analyzed with flow cytometry and immunocytochemistry. Treatment of retinal explants with minocycline reduced microglial activation and simultaneously significantly decreased cell viability and increased the presence of TUNEL-labeled cell profiles. This treatment also prevented the migration of microglial cells towards the outer nuclear layer, where cell death was most abundant. The LPS treatment increased microglial activation but had no effect on cell viability or microglial distribution. Finally, partial microglial removal with Lip-Clo diminished the cell viability in the retinal explants, showing a similar effect to that of minocycline. Hence, cell viability is diminished in retinal explants cultured *in vitro* when microglial cells are removed or their activation is inhibited, indicating a neurotrophic role for microglia in this system.

## Introduction

The accumulation and activation of microglial cells in the affected areas is a hallmark of retinal pathologies associated with apoptosis and retinal neuron degeneration [1, 2]. Microglial cells are absent from the Outer Nuclear Layer (ONL) in the normal retina [3] but are concentrated in the ONL when this layer is affected by pathological conditions [4–12].

Microglial cells may have either a neurotoxic (negative) or neurotrophic (positive) role in the degeneration process. In support of the neurotoxic role, several authors have reported that the number of degenerating cells in pathological retinas is reduced by the inhibition of microglial activation [13–17]. Further, *in vitro* experiments have revealed that the degeneration of photoreceptors is greater when the cells are cultured with activated microglia or in microglia-conditioned media [18–21]. In this respect, microglia are sensitive to alterations of the cell environment and release cytotoxic molecules that can propagate cell death [22–24], exacerbating the original damage. According to the above data, microglia appear to have a neurotoxic effect, and the inhibition of their activation would favor the retinal cell survival.

However, other studies have indicated that microglia have a positive effect on the survival of photoreceptor cells. That is, photoreceptor degeneration was found to be greater when the number of microglial cells was reduced by blocking stromal-derived factor-1, which stimulates the recruitment of macrophage/microglial cells to the retina [25]. Conversely, retinal degeneration was slowed and cone cell survival enhanced by the activation of retinal microglia through the systemic administration of granulocyte-colony stimulating factor and erythropoietin. [25]. Other studies have also reported that a reduction in microglial activation increases photoreceptor degeneration [26, 27]. Accordingly, microglia may exert a neurotrophic impact on retinal cells.

Therefore, the function of microglial cells during cell degeneration appears to be complex and modulated by age, the nature of the damaging stimulus, and the presence of external factors, among others [2, 28]. In retinal explants from *rd10* mice, which show inherited photoreceptor degeneration [29], photoreceptor death was diminished by the depletion of microglia and by treatment with insulin-like growth factor-1 (IGF-1); however, the neurotrophic effect of IGF-1 was significantly weaker in *rd10* explants after clodronate-induced microglial cell depletion [29]. Hence, microglial cells in these explants appear to be neurotoxic in the absence of IGF-1 but also play a key role in the full neurotrophic effect of this factor when present.

Retinal explants constitute a useful model for studying interactions between microglia and degenerating neurons. Also, they offer a system in which the cells are accessible to manipulation but maintain many of the extracellular features and cellular interactions of the *in vivo* situation. Organotypic culture of the retina can be considered a bridge between the dissociated cell culture, when the cells can be readily manipulated but are in a completely different environment, and the *in vivo* situation, in which cell manipulation is challenging. In addition, the explants enable the study of the microglial response without the influence of the blood-derived cells that also participate in the response to *in vivo* retinal degeneration [10] and modulate the microglial response [30].

Our aim was to exploit these advantages in studying the function of microglial cells in the retina. A previous study in our laboratory revealed that the mouse retinal cytoarchitecture is better preserved in explants from retinas at 10 postnatal days (P10) than at the adult stage, and that cell viability is higher in explants from developing than in adult retinas [31]. Although microglia become activated in explants of both ages, P10 was selected for the study because of the better preservation of normal architecture and greater viability.

Activation of the microglia was manipulated by culturing retinal explants with minocycline or lipopolysaccharide (LPS). Minocycline reduces microglial activation [32–34] and has been reported to boost the neuron survival in the CNS, exerting a neuroprotective effect in the brain [34–37] and retina [33, 38–42]. By contrast, other studies have reported that minocycline treatment worsened cell degeneration in some pathological situations [43–46]. LPS, a component of the outer cell wall of Gram-negative bacteria, has neurotoxic effects presumably mediated by microglia [22, 47–50] and increases microglial activation. In addition, microglial cells can be depleted from explants by treatment with clodronate-loaded liposomes (Lip-Clo). Lip-Clo selectively induce degeneration of macrophage/microglial cells as they are engulfed by phagocytic cells that degrade their lipid envelope, releasing their toxic content inside the phagocyte and causing its death; by contrast, non-phagocytic cells are not affected [51]. Treatment with Lip-Clo has been found to deplete microglial cells in explants of different brain regions [52, 53] and retina [29, 54].

According to our results, the viability of retinal cells in the explants is not affected by intensified microglial activation but is significantly diminished by significantly lower activation of microglial cells or their depletion, supporting the idea that microglial cells have a neuroprotective role in this system.

## Material and Methods

### Animals

C57BL/6 mice were provided by Harlan (Barcelona, Spain) through the Animal Experimentation Service of the University of Granada. Retinas were taken from 10-day-old (P10) animals after decapitation. Experimental procedures adhered to the ARVO Statement for the Use of Animals in Ophthalmic and Vision Research and were approved by the Research Ethics Committee of the University of Granada (Comité de Ética en Experimentación Animal, Permit Number: 2011–357), following the guidelines of the European Union Directive 2010/63/EU on the protection of animals used for scientific purposes.

### *In vitro* culture of retinal explants

The enucleated eyes were transferred to a Petri dish containing Gey’s balanced salt solution (Sigma, St. Louis, USA) supplemented with 5 mg/ml glucose (Sigma) and 50 IU-μg/ml penicillin-streptomycin (Invitrogen, Paisley, UK). Explants (about 3 mm in diameter) containing the central part of each retina were placed on membrane culture inserts (Millicell CM, Millipore, Bedford, MA, USA; pore size 0.4 μm) in 6-well plates (vitreal side downward) and cultured for two days in the culture medium under previously described conditions [31]).

### Treatments of retinal explants

The explant from the right retina of each animal was subjected to experimental treatment, while the left one served as control.

#### Minocycline treatment

The minocycline-treated explants were cultured for two days in medium containing 100 μg/ml (200 μM) minocycline hydrochloride (Sigma). Controls were cultured in normal culture medium.

#### LPS treatment

Explants subjected to LPS treatment were cultured for 24 h in normal medium; followed by another 24 h of culture in medium supplemented with LPS (from *E*. *coli* strain 0111:B4, Sigma) to a final concentration of 5 μg/ml. Control explants were cultured in normal medium for the second 24-h period.

#### Combined treatment with minocycline and LPS

Some explants were subjected to double treatment with minocycline and LPS. These explants were cultured for two days in medium containing minocycline hydrochloride (200 μM) supplemented with LPS (5 mg/ml) for the last 24 h of culture.

#### Lip-Clo treatment

The liposomes were supplied by ClodronateLiposomes.com (Amsterdam, The Netherlands). Freshly prepared retinal explants were incubated for 90 min in ice-cold Gey’s balanced salt solution supplemented with glucose and antibiotics containing Lip-Clo at a 1:5 (v/v) dilution. The explants were then placed on Millicell inserts and cultured for two days in normal medium. On the second culture day, the explants received a second dose of Lip-Clo, which was pipetted onto the explant surface.

Two types of control were used for the Lip-Clo treatment: untreated explants, and explants treated with PBS-containing liposomes (Lip-PBS).

### Determination of cell viability and analysis of microglial cell proliferation by flow cytometry

Cell viability was determined by flow cytometry of cell suspensions from dissociated retinal explants using fluorescein diacetate (FDA) and propidium iodide (PI) (both from Sigma), as previously reported [31]. The inclusion of cell fragments and cell aggregates was minimized by considering events with the size and granularity only of single cells (S1 Fig).

Flow cytometry was also used to quantify the percentage of CD11b-positive microglial cells showing Ki67 immunoreactivity. This percentage reflects the proliferative rate of microglia as the anti-Ki67 antibody recognizes a nuclear protein present in proliferating cells. Explants were fixed in 1% paraformaldehyde in PBS for 20–40 min, rinsed in PBS containing 0.1% Tween (Sigma), permeabilized for 10 min in PBS with 0.5% Triton X-100 (Sigma), dissociated and incubated in phycoerythrin-conjugated anti-CD11b antibody (Serotec, Oxford, UK; rat monoclonal antibody, clone 5C6; immunogen: thioglycollate-elicted peritoneal macrophages; dilution 1:25) and Alexa 488-conjugated anti-Ki67 antibody (BD Pharmingen, Franklin Lakes, NJ, USA; mouse monoclonal antibody, clone B56; immunogen: human Ki67; dilution 1:33). CD11b-positive and Ki67-positive cells were then quantified by flow cytometry using FACS Vantage and FACS Canto II flow cytometers (Becton Dickinson, Franklin Lakes, NJ, USA).

### Immunocytochemistry

Microglial cells and/or peripheral monocytes were identified by immunocytochemistry with either anti-CD11b (Serotec; rat monoclonal antibody, clone 5C6; immunogen: thioglycollate-elicited peritoneal macrophages; dilution 1:100) or anti-CD45 (Serotec, rat monoclonal antibody, clone IBL-3/16; immunogen; purified B cells from mouse lymph nodes; dilution 1:40), as previously reported [31]. Both antibodies show equivalent labeling of microglial cells in organotypic cultures of the mouse retina [31]. Counterstaining was done with Hoechst 33342 nuclear dye (Sigma).

Some sections were double-immunolabeled with either anti-CD11b or anti-CD45 and anti-Poly-ADP ribosylated (PAR) polymers (Alexis Biochemicals, San Diego, CA, USA; mouse monoclonal antibody, clone 10H; immunogen: purified Poly-ADP ribose; dilution 1:50) to identify activated microglial cells. PAR polymers are produced by the activity of the enzyme Poly-ADP Ribose Polymerase-1 (PARP-1), which is increased in activated microglia [55].

### TUNEL staining

Hydrated and permeabilized sections were treated for 1 h at 37°C with a solution containing 0.01 units/μl of terminal deoxynucleotidyl transferase enzyme (TdT) (Promega, Madison, WI, USA) in TdT buffer (pH 6.8) (Promega) and 3 nmol/ml of tetramethylrhodamine-dUTP (Roche Diagnostics, Mannheim, Germany). After incubation, sections were washed with PBS and counterstained with Hoechst 33342 (Sigma).

### Analysis of the production of TNF-α

The TNF-α content of culture media conditioned by explants treated with minocycline or LPS was analyzed with a commercial ELISA assay (Ray Bio mouse TNF-α ELISA kit, RayBiotech, Norcross, GA, USA). Optical density of each sample was read in a spectrophotometer microplate reader (Multiskan Ascent, Thermo Scientific, Madrid, Spain) using a 450-nm filter. The TNF-α content of media from untreated explants served as control.

### Imaging and analysis

Confocal images were obtained with a Leica TCS-SP5 microscope (Leica, Wetzlar, Germany). Findings reported by microscopic images were representative of observations performed in at least three explants.

Quantitative data were expressed as means ± SEM from at least five independent experiments. Statistical significance was determined using Student’s t-test.

## Results

### Decreased cell viability and increased presence of TUNEL-positive cells in minocycline-treated retina explants

We conducted a pilot study to determine whether the concentration of minocycline used was toxic to retinal cells in the explants, analyzing the influence of different concentrations of minocycline on the cell viability (S2 Fig). No significant differences in cell viability (determined by flow cytometry) were observed among explants treated for 2 days *in vitro* with minocycline concentrations ranging between 20 and 200 μM. A dose of 200 μM was selected for subsequent experiments, because it caused the sharpest reduction in microglial cell activation as measured by TNF-α production [56].

**Fig 1:**
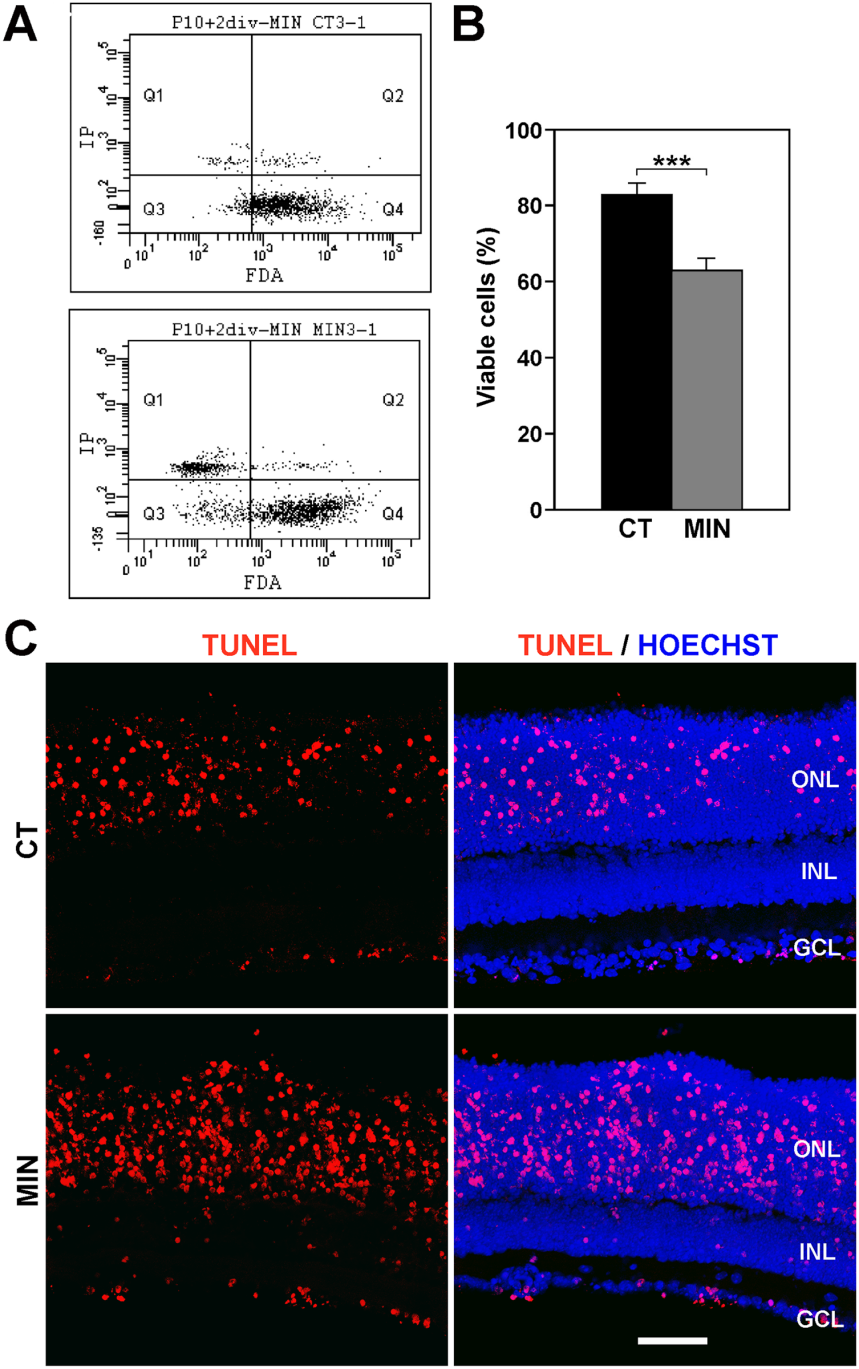
Treatment with minocycline reduces cell viability and increases the numbers of apoptotic cells. **A**. Flow cytometry dot plots of viable cells (stained with Fluorescein diacetate, FDA) and dead/dying cells (labeled with propidium iodine, IP) in a control explant (upper plot) and in an explant treated with minocycline (lower plot). **B**. Bar graph showing that the percentage of viable cells (±SEM) is ≈20% higher in control explants (CT, black) than in minocycline-treated ones (MIN, gray). Eight explants were analyzed per condition. *** denotes significant differences (P<0.001, Student’s t-test) between MIN and CT explants. **C**. TUNEL staining of control (CT, upper panels) and minocycline-treated explants (MIN, lower panels). The images on the left show the TUNEL signal (red), whereas those on the right also display the nuclei revealed with Hoechst staining (blue). Note that the presence of TUNEL-stained profiles, located mainly within the Outer Nuclear Layer (ONL), increases when explants are treated with minocycline. Representative images of at least three different explants per condition. INL, Inner Nuclear Layer; GCL, Ganglion Cell Layer. Scale bar, 50 μm.

The cell viability, as assessed by flow cytometry, was significantly lower in explants treated with 200 μM minocycline for 2 days *in vitro* than in untreated explants (Fig 1A and 1B), and TUNEL-labeled profiles were more frequently observed in the treated *vs*. untreated explants (Fig 1C). Therefore, the numbers of dying cells were increased in the retinal explants by the minocycline treatment.

### Decreased activation of microglial cells after minocycline treatment

**Fig 2:**
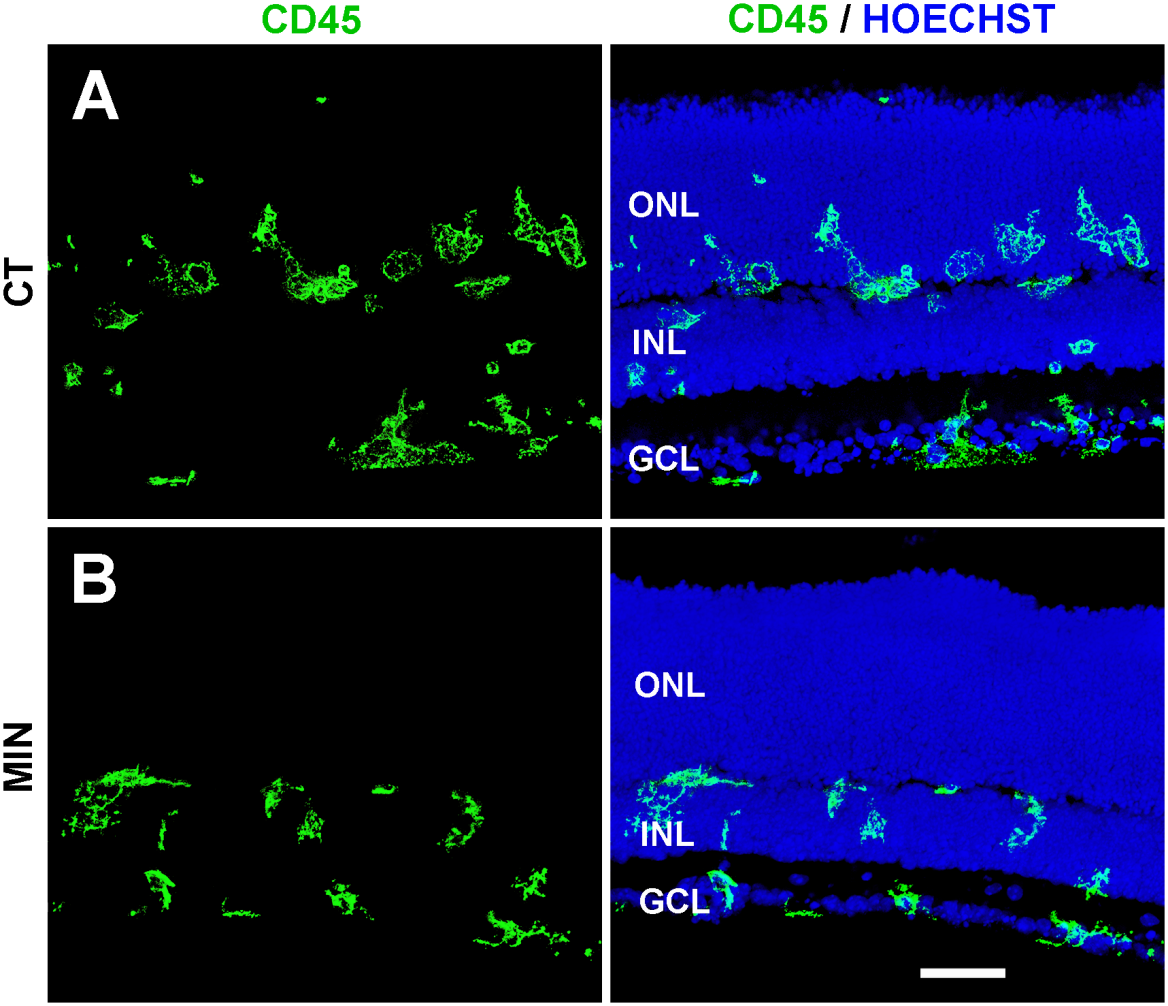
Microglial distribution in minocycline-treated and untreated explants. The left panel shows microglial cells (stained with anti-CD45 antibody, green), whereas the right panel depicts both microglial cells and the location of retinal layers (revealed by nuclear staining with Hoechst, blue). **A**. Microglial cells in the control explants (CT) are frequently seen within the Outer Nuclear Layer (ONL). **B**. In minocycline-treated explants (MIN), microglial cells are more compact and do not penetrate the ONL. Representative images of at least three different explants per condition. INL, Inner Nuclear Layer; GCL, Ganglion Cell Layer. Scale bar, 50 μm.

Minocycline treatment also modified the morphological features, distribution, and activation state of microglial cells. These had a honeycomb-like swollen appearance in untreated explants (Fig 2A) and acquired a more compact morphology in minocycline-treated explants (Fig 2B), suggesting a lower state of activation. Moreover, microglial cells entered the ONL in control explants but not in minocycline-treated explants (Fig 2A and 2B). Given that most cell death was detected at the ONL (see Fig 1C) and that a portion of dead/dying cells is reported to be engulfed by microglial cells in the ONL [31], minocycline treatment resulted in abundant non-phagocytosed debris (S3 Fig).

**Fig 3:**
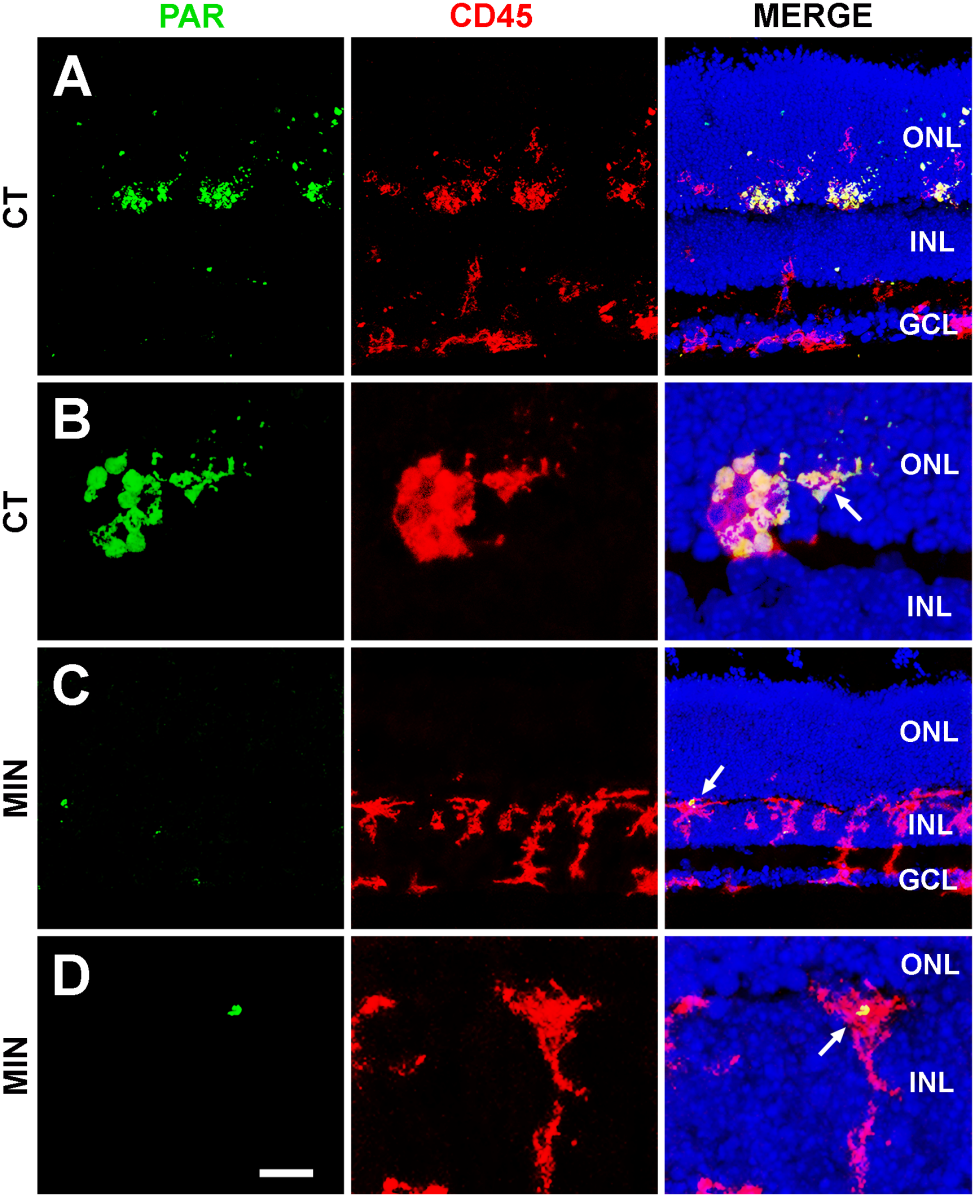
PARP-1 activity in untreated (A,B) and minocycline-treated (C,D) retinal explants. The left column shows the labeling with an antibody recognizing Poly-ADP-Ribose polymers (PAR, green) due to activity of the enzyme PARP-1. The central column exhibits microglial cells revealed by anti-CD45 antibody (red). Finally, pictures in the right column merge the two signals and that of Hoechst labeling of the nuclei. Note that minocycline-treatment (MIN) markedly reduces the PAR immunoreactivity of microglial cells in the control (CT) retinal explant. ONL, Outer Nuclear Layer; INL, Inner Nuclear Layer; GCL, Ganglion Nuclear Layer. Representative images of three retinal explants per treatment. Scale bar, 35 μm for A, 10 μm for B, 34 μm for C, and 9 μm for D.

PARP-1 activity was used to determine the effect of treatment on the activation state of microglial cells. Microglia showed strong PAR immunoreactivity in the ONL of untreated explants (Fig 3A and 3B), whereas scant PAR immunoreactivity was detected in minocycline-treated explants (Fig 3C and 3D), indicating less activation of microglial cells in the latter.

**Fig 4:**
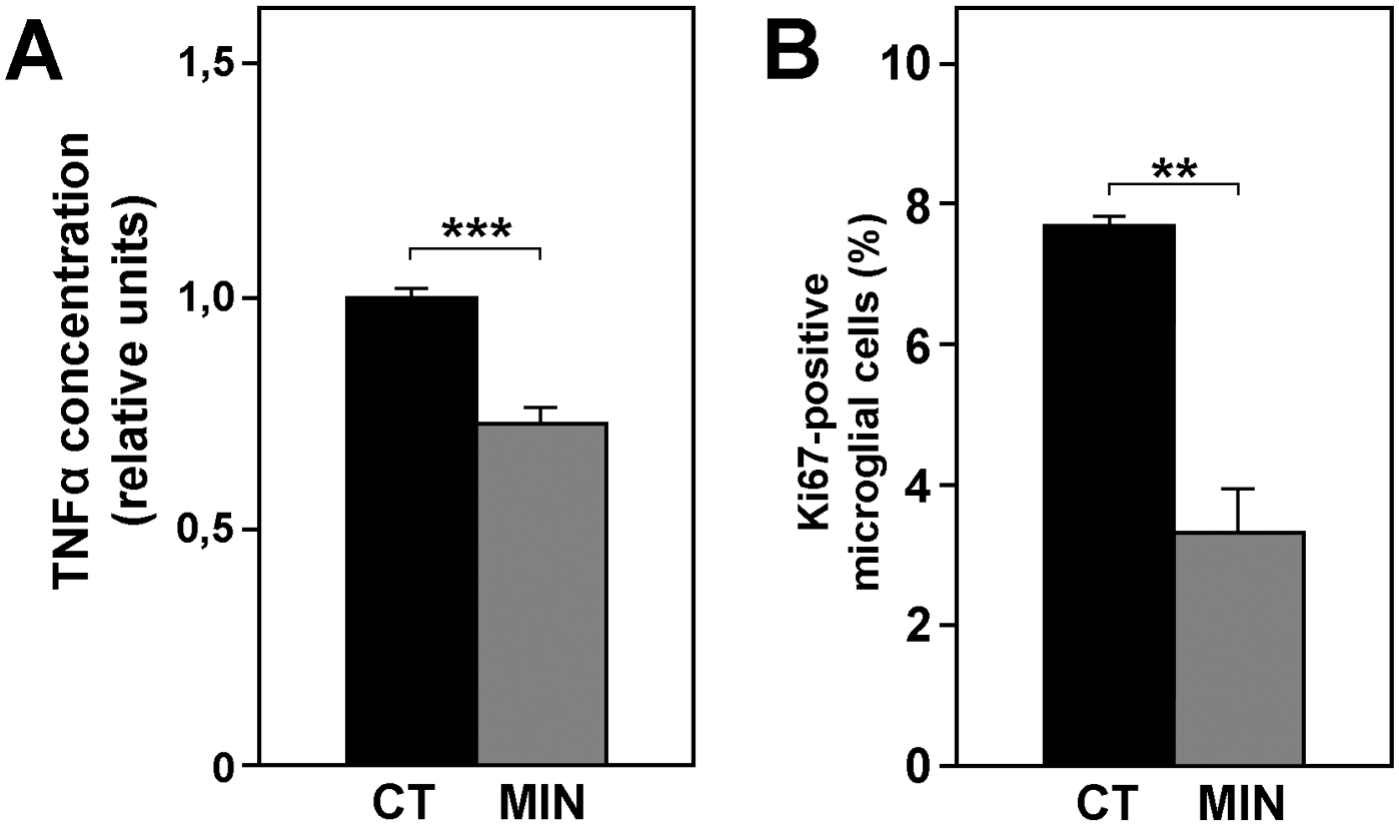
Minocycline treatment inhibits activation of microglia. The release of TNF-α (**A**) and the percentage of proliferating microglial cells (determined by anti-Ki67 labeling, **B**) decreased after incubation of retinal explants with minocycline. Bars represent mean values ± SEM of 12 experimental explants with their respective controls for A, and 5 for B. Asterisks indicate significant differences between the control (CT, black bars) and minocycline (MIN, gray bars) conditions (** P<0.01, *** P<0.001, Student's t-test).

Because TNF-α production is higher in activated microglia [57], the amount of TNF-α released into the culture medium can be used to estimate the activation level of microglia in cultured retinal explants. Our results show that TNF-α concentration was reduced by the minocycline treatment (Fig 4A), in agreement with previous data [58].

Finally, given that proliferative activity is increased in activated microglial cells [59], we studied the presence in the explants of Ki67 protein, a marker of proliferation, as an additional indicator of microglial activation. The proportion of proliferating microglial cells (CD11b-positive/Ki67-positive cells) in untreated explants, as determined by flow cytometry, was reduced by nearly 50% in minocycline-treated explants (Fig 4B).

Taken together, the above results confirm that the minocycline treatment effectively reduced the activation of microglial cells in the retinal explants.

### LPS treatment increases microglial activation but does not affect retinal cell viability

**Fig 5:**
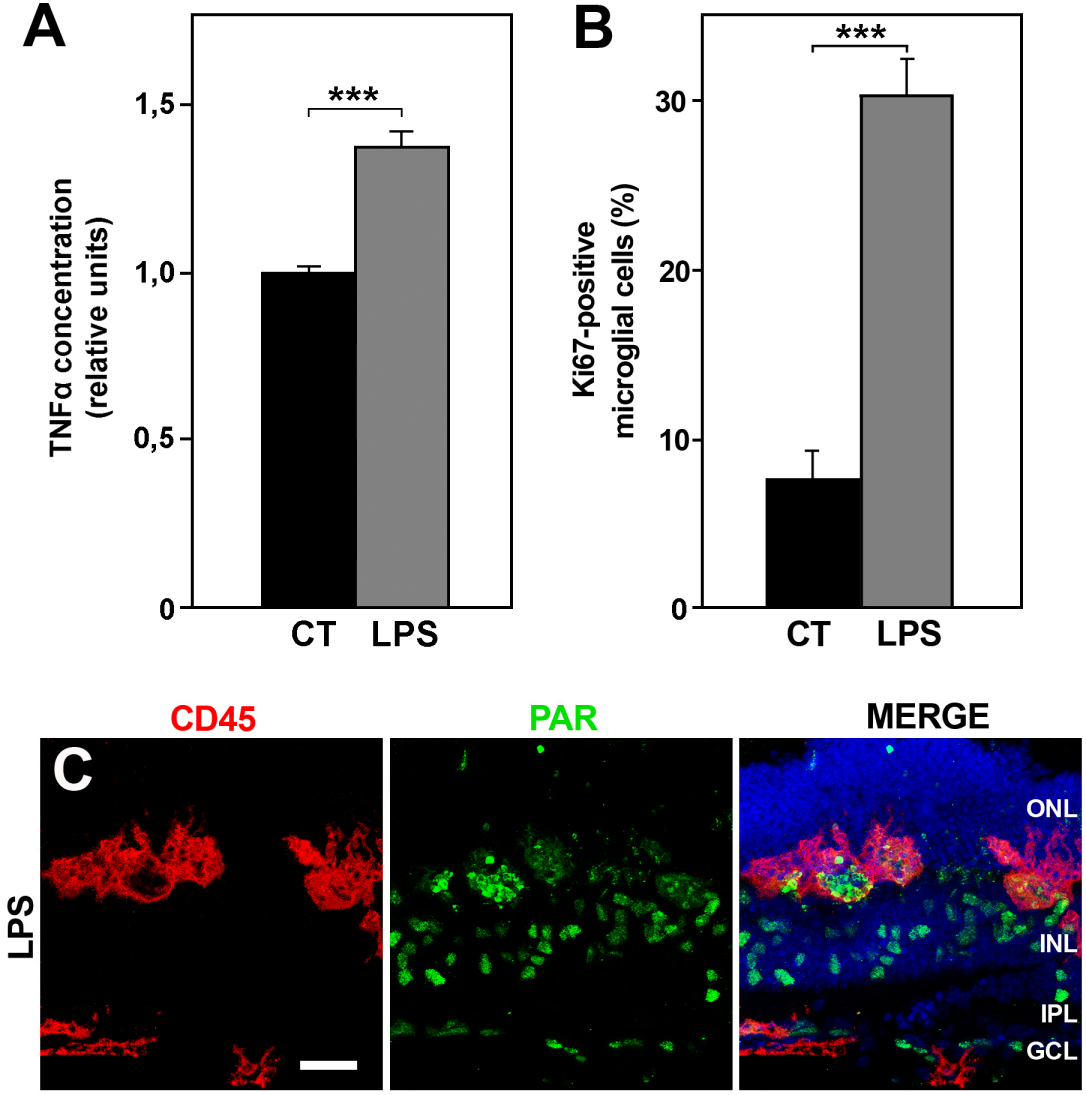
Effects of LPS treatment on retinal explants. **A**: The release of TNF-α was significantly increased in explants treated with LPS (LPS, gray bar) with respect to untreated explants (CT, black bar). Bars represent mean values of 12 explants per condition ± SEM. *** indicates significant differences (P<0.001, Student's t-test). **B**: Incubation with LPS (LPS, gray bar) also greatly augmented the proportion of proliferating microglial cells (labeled with anti-CD11b and anti-Ki67) with respect to control explants (CT, black bar). Bars represent mean percentages of 5 explants per condition ± SEM; *** indicates significant differences (P<0.001, Student's t-test). **C**: The PAR immunostaining (green) in LPS explants was similar to that observed in the untreated explants. Pictures in C are representative of 3 explants per condition. ONL, Outer Nuclear Layer; INL, Inner Nuclear Layer; IPL, Inner Plexiform Layer; GCL, Ganglion Cell Layer. Scale bar, 30 μm.

**Fig 6:**
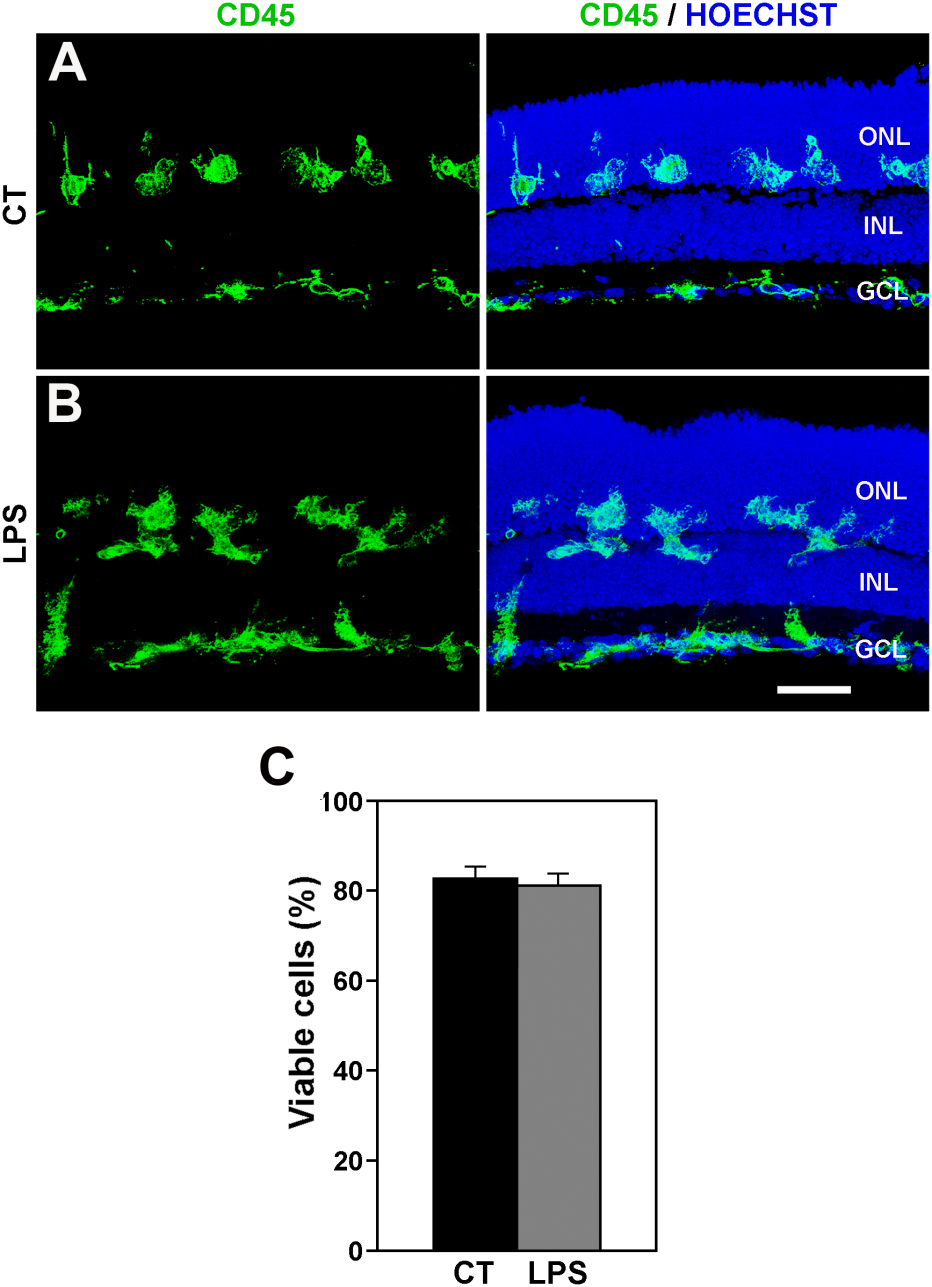
LPS treatment does not modify the distribution of microglial cell and cell viability. **A, B**: Distribution of microglial cells in control (A, CT) and LPS-treated (B, LPS) retinal explants. Left panels display microglial cells labeled with anti-CD45 antibody, while right panels also show cell nuclei stained with Hoechst. The distribution of microglia in LPS-treated explants is similar to that of untreated explants. Representative images of 3 different explants per condition. Scale bar, 50 μm. **C**: Bar graph showing that LPS treatment has no significant effect on cell viability in retinal explants. Bars represent mean values ± SEM of 5 explants).

Incubation of retinal explants in the presence of LPS induced a greater release of TNF-α in the culture media (Fig 5A) and augmented the proportion of proliferating (Ki67-positive) microglia (Fig 5B). These results support the view that treatment with LPS increases the activation state of microglial cells in the retinal explants. By contrast, immunocytochemical staining with anti-PAR antibody and the activated appearance of microglial cells were similar to that seen in control explants (Fig 5C and 5D; compare with Fig 3). In addition, LPS treatment had no appreciable effect on the distribution of microglial cells, which were located in all retinal layers, including the ONL, in both LPS-treated and non-treated retinal explants (Fig 6A and 6B). Therefore, the treatment with LPS apparently heightened the activation state of microglial cells which was already present, although at a lower level, in non-treated retinal explants. This higher activation level of microglial cells did not affect the cell viability in the LPS-treated explants (Fig 6C).

**Fig 7:**
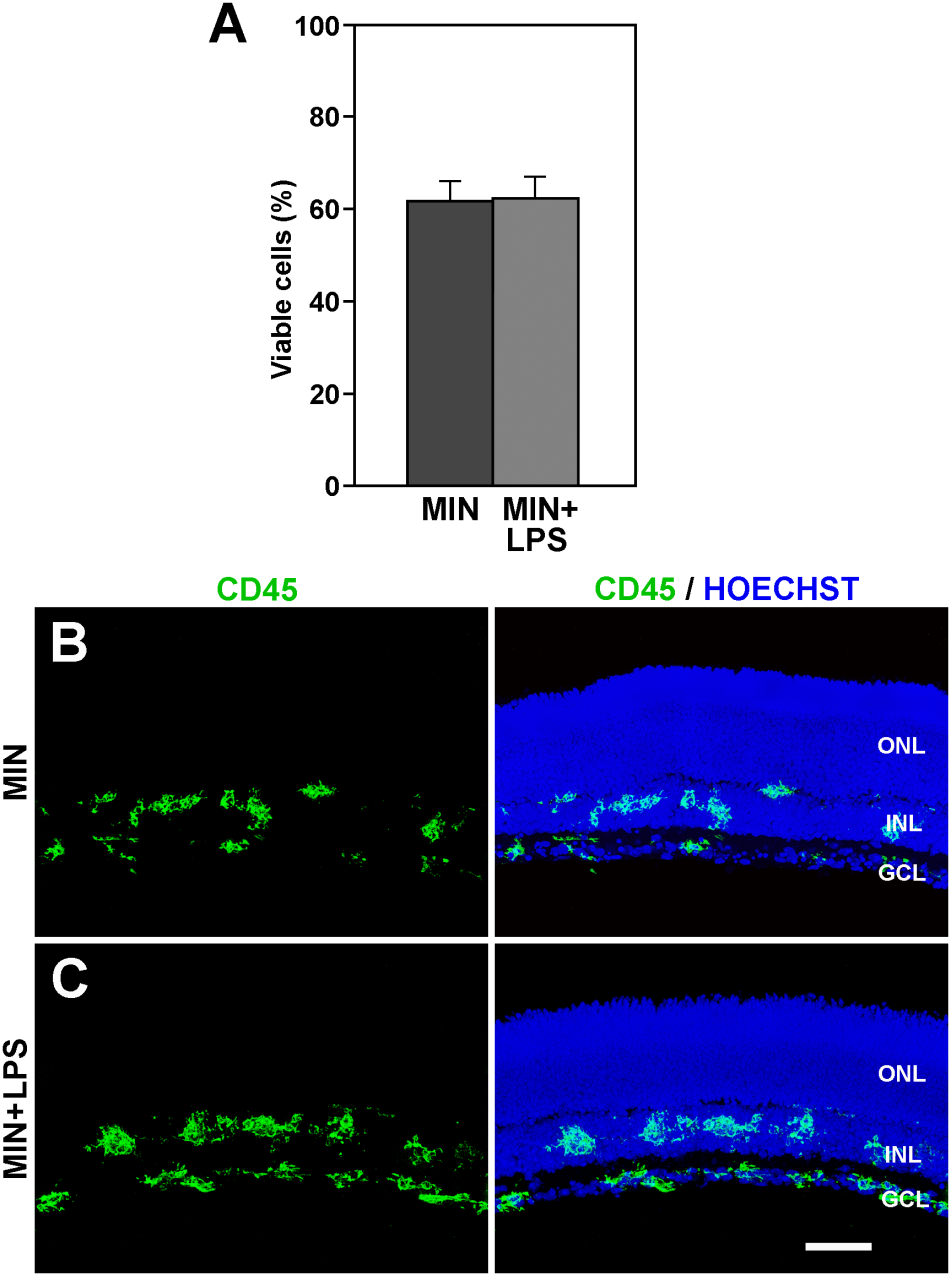
The effects of minocycline overcome these of LPS treatment. **A**: The cell viability in the explants treated with minocycline and LPS (MIN+LPS) is similar to that of explants treated with minocycline alone (MIN). Bars represent mean values ± SEM of 5 explants per condition. **B, C**: The distribution of microglial cells, as revealed by CD45 immunolabeling (green), is similar between explants treated with minocycline alone (MIN, B) and those treated with minocycline plus LPS (MIN+LPS, C). In addition to microglial cells, images in the right column also show the retinal layers revealed by the Hoechst staining of nuclei. Each image is representative of 3 explants. ONL, Outer Nuclear Layer; INL, Inner Nuclear Layer; GCL, Ganglion Cell Layer. Scale bar, 50 μm.

An important observation was that the fall in cell viability observed in explants treated with minocycline was indistinguishable from that found in explants exposed to double treatment with minocycline and LPS (Fig 7A). Microglial cells in the double minocycline/LPS-treated explants showed similar morphological features and distribution to those in the explants treated exclusively with minocycline (Fig 7B and 7C). Therefore, the anti-microglial activation effect of minocycline appeared to be stronger than the pro-microglial activation effect of LPS and was able to maintain a lower level of microglial activation.

### Lower cell viability after depletion of microglia with Lip-Clo

**Fig 8:**
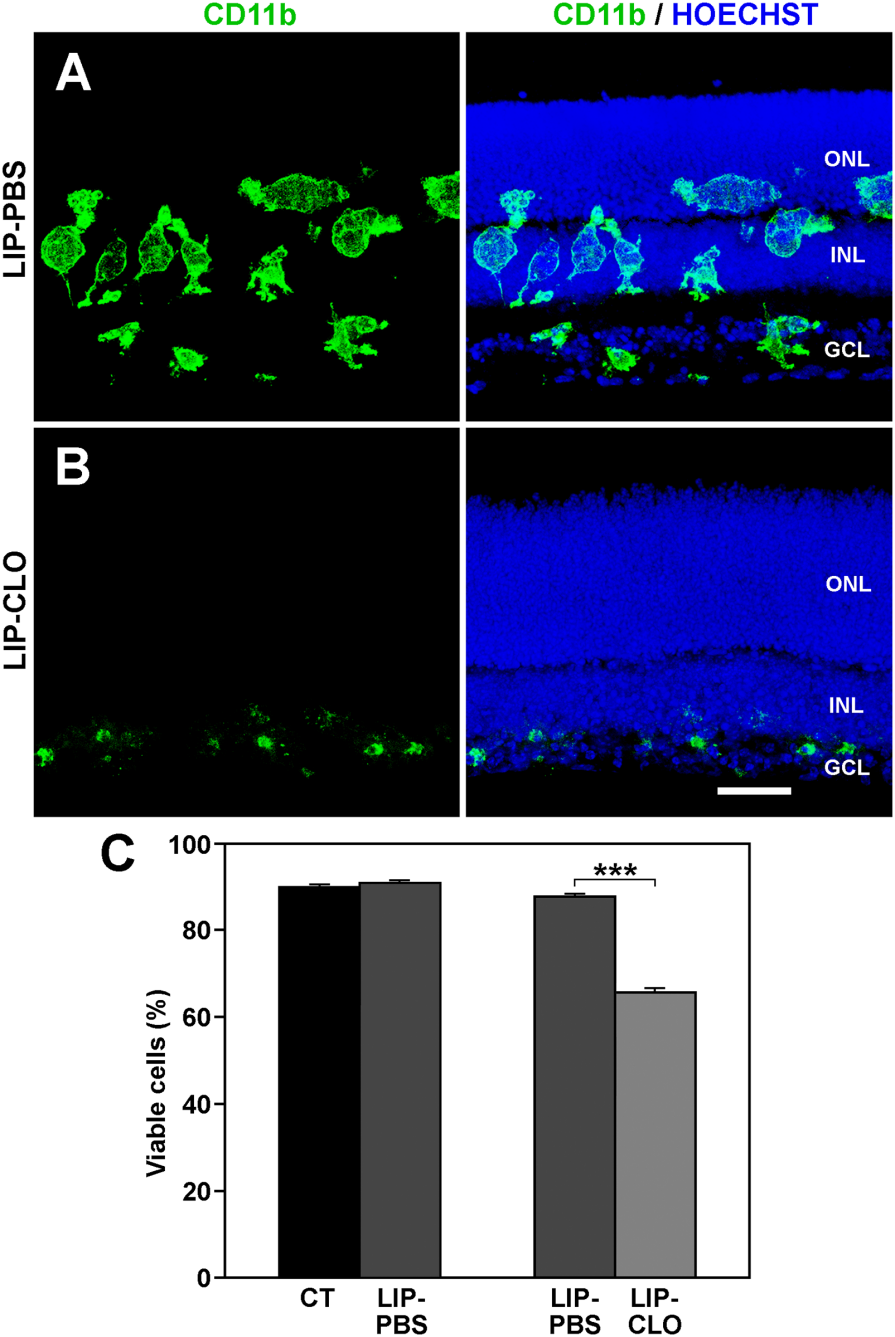
Effect of microglia depletion on cell viability in the retinal explants. **A**: The distribution of microglial cells, revealed by CD11b immunolabeling, in explants treated with liposomes loaded with PBS (LIP-PBS) is similar to that in control explants (compare with Figs 2A and 6A). The microglial cells have a swollen morphology, apparently due to the phagocytosis of liposomes. **B**: Few microglial cells are present in the retinal explants after treatment with clodronate-loaded liposomes (LIP-CLO). Note that the Outer Nuclear Layer (ONL) and Inner Nuclear Layer (INL) do not contain microglial cells and that the only positive signal appears in the most vitreal part of the retina (Ganglion Cell Layer, GCL). Besides the microglial marker, the right column also shows Hoechst staining to locate the retinal layers. Images in A and B are representative of three explants. Scale bar, 40 μm. **C**: The cell viability observed in LIP-PBS (dark-gray bars) is comparable to that observed in untreated explants (CT, black bar). However, the viability decreases by around 20% when explants are treated with LIP-CLO (LIP-CLO, light-gray bar). Bars represent mean values ± SEM of 5 explants per condition. *** denotes significant differences (P<0.001, Student’s t-test).

It could not be ruled out that the lower cell viability after minocycline treatment of the explants was due to a direct toxic effect of the drug on the retinal cells. It was therefore tested whether the microglial cells had a central role in diminishing cell viability by depleting them through the treatment of retinal explants with Lip-Clo. The effectiveness of the microglial depletion was demonstrated by immunocytochemistry. Explants treated with Lip-PBS contained numerous microglial cells with an activated morphology and a similar distribution to that in the untreated explants (Fig 8A). Treatment with Lip-Clo effectively eliminated most of the microglia, which were reduced to small cells or cell fragments at the vitreal border of the explants (Fig 8B).

Because the cell viability was similar between Lip-PBS-treated and untreated explants (Fig 8C, left bars), Lip-PBS-treated explants were used as controls of the Lip-Clo treatment. Microglia depletion in the Lip-Clo-treated explants induced a fall in cell viability in comparison to Lip-PBS-treated explants (Fig 8C, right bars), comparable to the drop in cell viability observed after the minocycline treatment of explants (compare Fig 1A with Fig 8C). These data suggest that the decline in cell viability after minocycline treatment of the explants was due to the change in microglial activation state rather than to a direct action of the antibiotic on the retinal cells.

## Discussion

The model of organotypic cultures of postnatal mouse retinal explants used in this study enabled us to determine whether the alteration of the activation state of microglia affected the viability/death of cells in the explants. We found that inhibition of microglial activation by minocycline treatment reduced cell viability and increased the presence of TUNEL-positive cells. In addition, cell viability also decreased when microglial cells were depleted with Lip-Clo. In contrast, greater microglial activation by LPS treatment had no appreciable effect on cell viability.

However, the lower cell viability found in explants treated with minocycline or Lip-Clo might be due to a decreased rate of phagocytosis by microglial cells. The consequent accumulation of non-phagocytosed cell debris, mostly FDA-negative, would diminish the proportion of viable FDA-positive cells, resulting in an apparent decrease in cell viability. Accordingly, the reduced cell viability observed was apparently related to an accumulation of fragments from dead cells rather than to greater cell death. We addressed this issue by limiting the flow cytometry analysis to particles with the size of whole cells, thereby avoiding the inclusion of cell fragments (which would be the entities to be phagocytosed) in order to record the true changes in cell viability regardless of the phagocytosis rate of the apoptotic fragments.

### Microglial activation in retinal explants

The preparation of retinal explants and their subsequent *in vitro* culture induced the activation of microglial cells [31]. The factors responsible for this reaction include the optic nerve transection, culture conditions, and absence of incoming blood-derived cells. Microglial cells are activated in response to the transection of the optic nerve performed during isolation of the retina for the explant preparation [60, 61]. Microglial cells also react to altered conditions during the culture. Studies in retinal explants of quail-embryo retina have reported superior preservation of the physiological characteristics of microglial cells when 25% horse serum was added to the basal medium with Earle’s salts [62], as in the present study. Finally, retinal explants preclude entry into the retina of monocytes/macrophages from the bloodstream. This absence of cells of blood origin may affect the activation state of microglia in the retina, given that blood-derived macrophages has been reported to modulate the response of microglia [30].

Our results verify that microglial activation is reduced by minocycline treatment of retinal explants, as evidenced by the reduction observed in TNF-α release, PAR immunoreactivity, and proliferation of the microglial cells. Minocycline treatment was also found to markedly impair the ability of microglial cells to migrate towards the ONL, despite that most cell death occurs in this layer.

### Neuroprotective effect of activated microglia in retinal explants

As stated in the Introduction, it has been consistently reported that microglial activation inhibition with minocycline reduces neuronal cell death in degeneration paradigms in the brain and retina. Therefore, treatments that lower the activation level of microglia could be expected to improve the survival of cells in the retinal explants. Surprisingly, our results indicated the opposite effect, given that the cell survival was lower and cell death higher with a reduction in microglial activation after minocycline treatment. Similar results, with effective inhibition of microglial activation and reduction of cell viability, have been described after prolonged minocycline treatment of organotypic cultures of spinal cord from neonatal rats [63].

The present results were repeated after partial removal of microglia in retinal explants by Lip-Clo treatment. It could be argued that dead microglia after Lip-Clo phagocytosis can release clodronate and cytotoxic factors into the medium and cause changes in cell viability of retinal explants. Although this possibility exists, previous *in vitro* studies using microglial depletion with Lip-Clo have demonstrated that cells grow in number [52] or are not apparently affected [53, 64] in different model systems. Furthermore, cell degeneration caused by factors released from dead microglia would also be irrelevant in our model system.

Our findings regarding lower cell viability after the inhibition of microglial activation or partial microglial removal in retinal explants agree with reports that microglial cells promote cell survival in certain situations and can therefore be considered neuroprotective [53, 65–70]. In this respect, it has been reported that the inactivation of microglia with minocycline increase neuron apoptosis in the developing brain [71], diminish the formation of precursors derived from Müller cells in the chick retina [72], and inhibit neurogenesis and oligodendrogenesis in the subventricular zone of postnatal rats [58].

The function of microglia in the retinal explants may depend on prior conditions. In this context, preconditioning of the CNS with potentially damaging stimuli (e.g. brief LPS treatment or hypothermia) was reported to protect against the effect of subsequent injuries [73, 74]. This effect appears to be mediated by the microglial response and is blocked by minocycline treatment [75]. In addition, the effects of microglial activation in hippocampal slices have been found to depend on the type of LPS treatment administered, with acute treatment prompting a neurotoxic activation of microglia and chronic treatment favoring a neuroprotective activation [76].

Hence, several factors affecting retinal microglia during the preparation and culture of retinal explants may induce the acquisition of a neuroprotective phenotype by these cells.

### Mechanisms accounting for the neuroprotective effect of microglial cells

Two main mechanisms underlie the neuroprotective effects of activated microglia: the release of neurotrophic factors and phagocytic activity. However, it cannot be ruled out that microglia may increase cell viability by other means.

Activated microglia produce neurotrophic molecules such as NGF, GDNF, and BDNF [77–80], and therefore the inhibition of microglial activation may reduce the release of these cell-survival-sustaining factors. It has been reported [71] that the prevention of microglial activation induces the death of layer V neurons of the postnatal mouse cortex, apparently related to the decrease in IGF-1 production by microglial cells.

The pro-survival effect of the molecules released by microglia may also affect a third cell type that in turn has a neuroprotective effect on neural cells. Thus, factors released by activated microglia in the retina may influence the response of Müller cells, thereby contributing to protect photoreceptor cells [79]. In this sense, Müller cells previously co-cultured with activated microglia were found to boost the survival rate of a photoreceptor cell line exposed to oxidative stress [27]. There is also some evidence that the presence of microglia may be required for some factors to exert their neurotrophic effect, although microglia would not be involved in their production. As noted above, IGF-1 treatment reduces the cell death found in retinal explants from *rd10* mutant mice, but it has been demonstrated that the depletion of microglia from these explants diminishes the neurotrophic effect of IGF-1 treatment [29].

The neuroprotective role of microglia may also be related to their phagocytic activity. Cell death is known to propagate from dead/dying photoreceptors among nearby photoreceptors in a so-called “bystander effect”, which would release noxious factors that damage additional photoreceptors [81, 82]. Thus, the release of abundant toxic factors may result from a reduction in the phagocytic activity of microglial cells that remove the dead/dying cells. In explants, the detrimental effect of minocycline treatment would be more marked than in *in situ* retinas, in which blood-borne macrophages would cooperate with retinal microglia to remove cell debris [10]. It has been noted that photoreceptor degeneration worsens when the recruitment of bone marrow macrophages to the retina is blocked [25], i.e. when the number of external phagocytes is reduced. Therefore, the amount of cell debris removed by the phagocytic activity of retinal microglia would inversely correlate with the amount of cell death in the explants, and microglial depletion or the inhibition of microglia activation would increase cell death. This is consistent with findings that microglial phagocytosis is needed for the effective removal of potentially noxious degenerated tissue in order to maintain the normal state of nervous tissue [83, 84].

In summary, the present findings reveal that microglial cells in retinal explants cultured *in vitro* favor the viability of neural cells. Investigation of the mechanisms involved in this neuroprotective outcome may be relevant in formulating paradigms to maintain *ex vivo* retinal tissue in studies on microglia as a therapeutic target in retinal pathologies.

## Supporting Information

S1 Fig Dot plot showing all events resulting from the dissociation of a retinal explant. The analysis was restricted to events (outlined area) with the size (FSC-H) and granularity (SSC-H) of single cells. The area containing single cells was established with fluorescent beads of known size (8–17 μm) and granularity. Events with values 1.5-fold higher or lower than the expected ones were considered to be cell fragments (on left) or cell aggregates (on right), respectively, and not included in the analysis.
(PDF)

S2 Fig Concentrations of minocycline between 20 and 200 μM showed similar effects on the viability of cells in the explants. Data are mean values ± SEM of three different explants for each concentration.
(PDF)

S3 Fig Microglial cells (anti-CD11b, green) did not phagocytose the numerous TUNEL-labeled cells (red) that appeared in the Outer Nuclear Layer (ONL). INL, Inner Nuclear Layer; GCL, Ganglion Cell Layer. Scale bar, 50 μm.
(PDF)
