# Supplementary figures and images for: Microglial Activation Promotes Cell Survival in Organotypic Cultures of Postnatal Mouse Retinal Explants
Source: PLoS One. 2015 Aug 7;10(8):e0135238. doi: 10.1371/journal.pone.0135238 (PMC4529135; doi:10.1371/journal.pone.0135238)

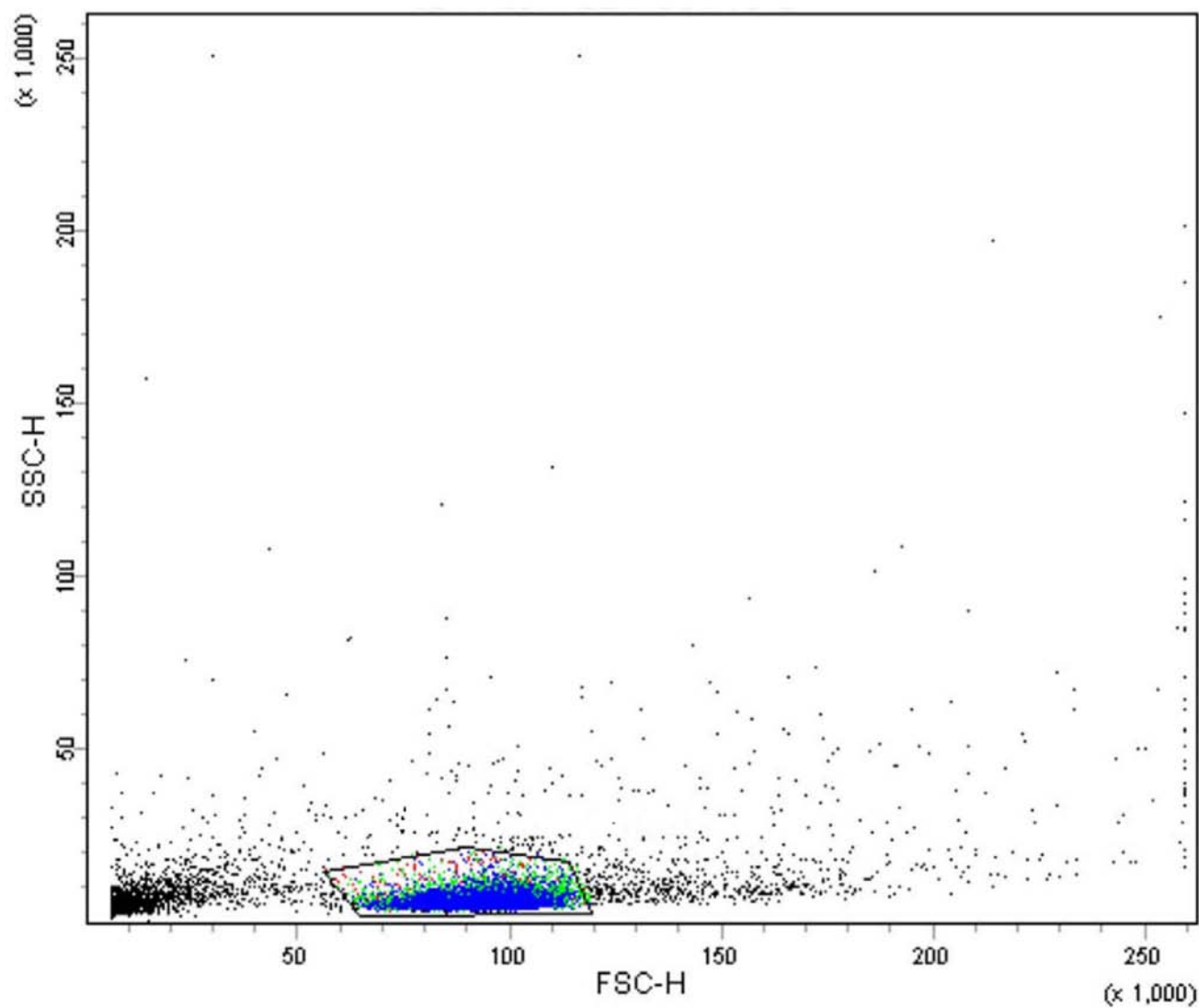

Supplement: S1 Fig — The analysis was restricted to events (outlined area) with the size (FSC-H) and granularity (SSC-H) of single cells. The area containing single cells was established with fluorescent beads of known size (8–17 μm) and granularity. Events with values 1.5-fold higher or lower than the expected ones were considered to be cell fragments (on left) or cell aggregates (on right), respectively, and not included in the analysis. (PDF) [file pone.0135238.s001.pdf]

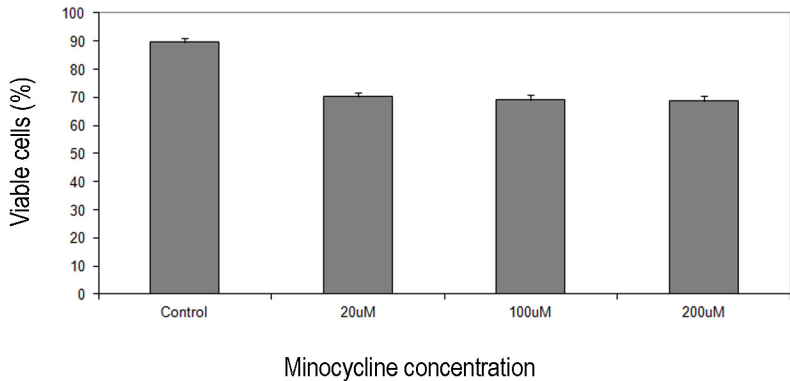

Supplement: S2 Fig — Data are mean values ± SEM of three different explants for each concentration. (PDF) [file pone.0135238.s002.pdf]

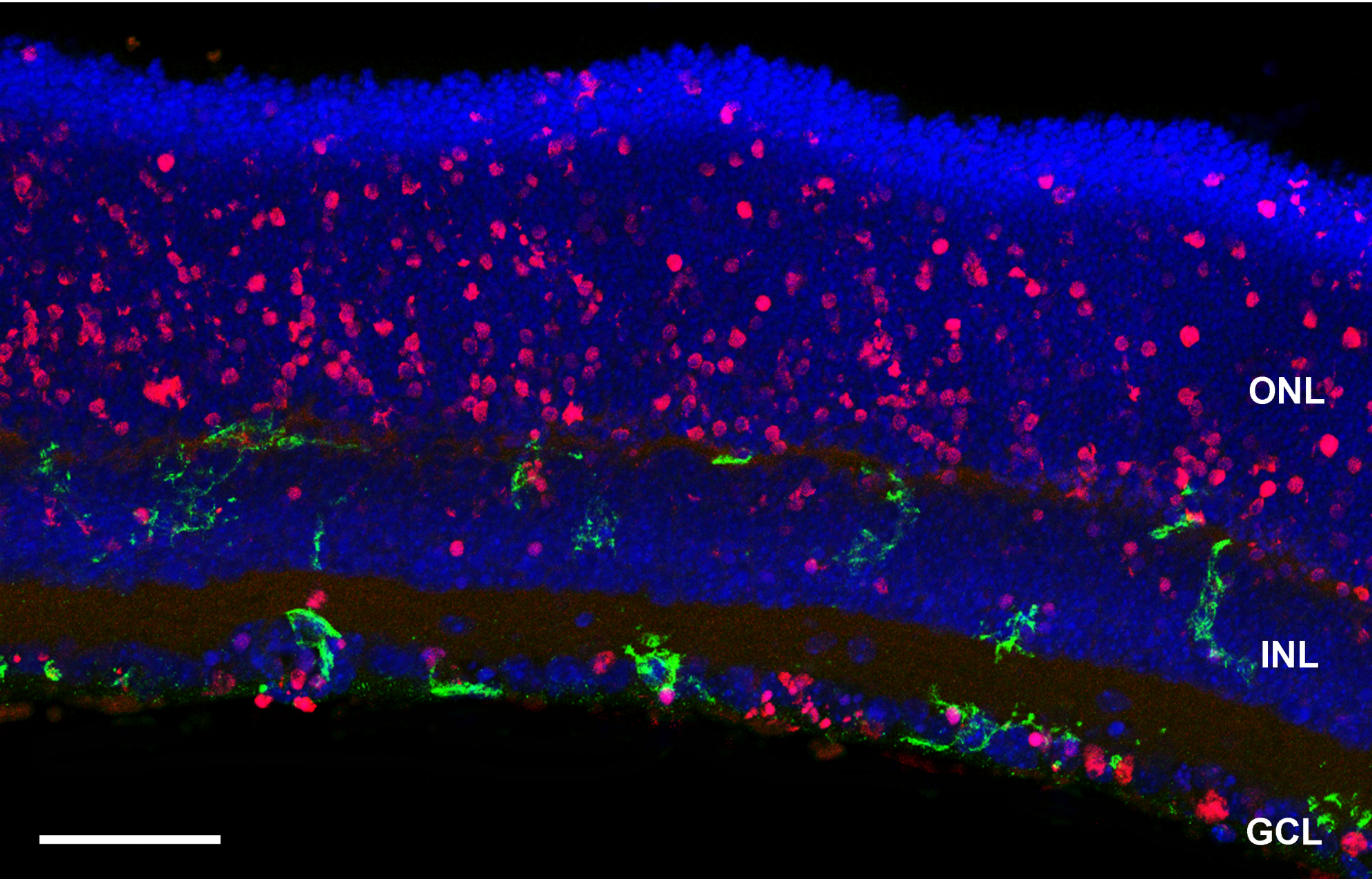

ONL

INL

GCL

Supplement: S3 Fig — INL, Inner Nuclear Layer; GCL, Ganglion Cell Layer. Scale bar, 50 μm. (PDF) [file pone.0135238.s003.pdf]
